# Association and temporal sequence of pneumonia and gastrointestinal bleeding after acute ischemic stroke

**DOI:** 10.1186/s12876-024-03312-w

**Published:** 2024-07-05

**Authors:** Runhua Zhang, Huiqing Hou, Xingquan Zhao, Liping Liu, Yilong Wang, Gaigen Liu, Yongjun Wang, Ruijun Ji

**Affiliations:** 1 Beijing Tiantan Hospital, Capital Medical University, Beijing, China; 2 China National Clinical Research Center for Neurological Diseases, Beijing, China; 3 Center of Stroke, Beijing Institute for Brain Disorders, Beijing, China

**Keywords:** Pneumonia, Gastrointestinal bleeding, Stroke, Temporal sequence

## Abstract

**Background:**

Stroke-associated pneumonia (SAP) and gastrointestinal bleeding (GIB) are common medical complications after stroke. The previous study suggested a strong association between SAP and GIB after stroke. However, little is known about the time sequence of SAP and GIB. In the present study, we aimed to verify the association and clarify the temporal sequence of SAP and GIB after ischemic stroke.

**Methods:**

Patients with ischemic stroke from in-hospital Medical Complication after Acute Stroke study were analyzed. Data on occurrences of SAP and GIB during hospitalization and the intervals from stroke onset to diagnosis of SAP and GIB were collected. Multiple logistic regression was used to evaluate the association between SAP and GIB. Kruskal-Wallis test was used to compare the time intervals from stroke onset to diagnosis of SAP and GIB.

**Results:**

A total of 1129 patients with ischemic stroke were included. The median length of hospitalization was 14 days. Overall, 86 patients (7.6%; 95% CI, 6.1-9.2%) developed SAP and 47 patients (4.3%; 95% CI, 3.0-5.3%) developed GIB during hospitalization. After adjusting potential confounders, SAP was significantly associated with the development of GIB after ischemic stroke (OR = 5.13; 95% CI, 2.02-13.00; *P* < 0.001). The median time from stroke onset to diagnosis of SAP was shorter than that of GIB after ischemic stroke (4 days vs. 5 days; *P* = 0.039).

**Conclusions:**

SAP was associated with GIB after ischemic stroke, and the onset time of SAP was earlier than that of GIB. It is imperative to take precautions to prevent GIB in stroke patients with SAP.

## Introduction

Medical complications are frequent among patients after ischemic stroke or hemorrhagic stroke. They can prolong length of hospitalization and increase the costs of care [1–3]. A lot of studies have revealed that the medical complications after stroke were associated with a poor prognosis [4–8]. The complications after stroke can hinder neurological recovery and significantly increase the odds of death [4, 5, 8]. Of the post-stroke complications, pneumonia is one of the most common medical complications with a prevalence of 10% in patients after stroke [9]. Stroke-associated pneumonia (SAP) can increase a higher risk of mortality at discharge, 90 days, and 1 year [10]. Gastrointestinal bleeding (GIB), as another common post-stroke complication, is a systemic complication. The frequency of GIB is varied considerably from 1.2 to 8.0% [11–14]. GIB may affect the therapy for acute ischemic stroke (AIS) such as antiplatelet or anticoagulant treatments [13]. Additionally, GIB can increase the risk of severe dependence and long-term mortality [11].

Despite the well-documented association between post-stroke complications and outcome, little is known about the interrelationship between the post-stroke complications. In our previous study, we found that SAP was significantly correlated with non-pneumonia medical complications after AIS [15]. Especially, compared with patients without SAP, the odds ratio of development of GIB in patients with SAP was 8.35 (95% CI 6.27–11.1) after adjusted for potential covariates [15]. However, the temporal sequence of these medical complications after AIS is unclear. Better understanding the characteristics of the complication after stroke can timely identified patients with a higher risk and take measures to prevent the subsequent complications. In the present study, we aimed to (1) evaluate the incidence of SAP and GIB, (2) verify the association between SAP and GIB, and (3) clarify the temporal sequence of SAP and GIB.

## Method

### Study patients

All patients with AIS registered in in-hospital Medical Complication after Acute Stroke (iMCAS) were included in this study. iMCAS is a prospective cohort study which is designed to (1) compare the risk of medical complications by stroke subtypes, (2) investigate the potential interrelationship between common in-hospital medical complications after stroke and (3) explore bio-markers or neuroimaging-markers for post-stroke medical complications. Patients admitted into the department of neurology at Beijing Tiantan hospital from May 2014 to May 2016 were consecutively registered in the iMCAS. Patients were eligible in this study if they fulfilled the following criteria: (1) age 18 or older; (2) hospitalized with a diagnosis of AIS according to the World Health Organization criteria; (3) confirmed by head computerized tomography and /or brain magnetic resonance imaging; (4) time from stroke onset to hospital admission within 7 days.

### Data collection and definitions

Data were collected by trained research coordinators using standardized electronic Case Report Form. The following data were documented through medical records or face-to-face interview: demographics (age, gender), stroke risk factors (hypertension, diabetes mellitus, dyslipidemia, atrial fibrillation, coronary heart disease, history of stroke or transient ischemic attack, and current smoking), history of using antiplatelet (aspirin, clopidogrel) and anticoagulation (warfarin), comorbidities (valvular heart disease, chronic obstructive pulmonary disease, peripheral artery disease, hepatic cirrhosis, peptic ulcer, renal failure, arthritis and cancer), prestroke dependence (modified Rankin Scale score [mRS] ≥ 3), the National Institutes of Health Stroke Scale (NIHSS) score at admission, in-hospital interventions (antiplatelet therapy within 48 h and recombinant tissue plasminogen activator), complications in hospital, length of hospitalization, and death in hospital. Additionally, time intervals from stroke onset to the diagnosis of SAP and GIB were calculated.

SAP and GIB were defined as previous studies [11, 15–17]. SAP was defined as clinical and laboratory indices of respiratory tract infection (fever, cough, auscultatory respiratory crackles, new purulent sputum, or positive sputum culture), and supported by typical chest X-ray findings. GIB was defined as clinical (any episode of fresh blood or coffee ground emesis, hematemesis, melena, or hematochezia), laboratory or radiographic evidence of gastrointestinal bleeding.

### Statistics analysis

We used mean and standard deviation (SD) or median and interquartile range (IQR) to describe characteristics for continuous variables, and percentages for categorical variables. According to the presence of GIB, the patients were categorized into two groups. Chi-square test, Student t test or Mann-Whitney U test was applied to test the difference between groups. To estimate the incidence of SAP and GIB, the number of patients diagnosed with SAP or GIB was as numerator and the number of included patients was as denominator. We used a series of logistic regression models to examine the association between SAP and GIB. In the model 1, no covariate was adjusted. In the model 2, we adjusted age, gender, hypertension, diabetes, dyslipidemia, atrial fibrillation, smoking, prestroke dependence. In the model 3, we adjusted the covariates in model 2 plus NIHSS score, hepatic cirrhosis, peptic ulcer, and cancer. In model 4, we adjusted the covariates in model 3 plus pre-hospital antiplatelet drug, pre-hospital anticoagulant, antiplatelet therapy within 48 h, intravenous thrombolysis, and length of hospitalization. Considering the non-normal distribution of timing, Kruskal-Wallis test was used to compare the timing from SAP and GIB. Additionally, a stratified analysis by stroke severity (NIHSS < 16 vs. NIHSS ≥ 16) and infarct location (posterior circulation infarct vs. non-posterior circulation infarct) was performed.

## Results

### Baseline characteristics and incidence of SAP and GIB

Table 1 presents the baseline characteristics of patients. From May 2014 to May 2016, a total of 1129 patients with ischemic stroke were enrolled in the study. The mean age was 58.7 ± 2.5 and 899 (79.6%) were male. The median length of hospitalization was 14 days (IQR 11–16). Compared with the patients without GIB, the patients experiencing GIB were more likely to be with a history of atrial fibrillation (17.0% vs. 5.6%, *P* = 0.001), higher NIHSS score (13 vs. 4, *P* < 0.001), have longer hospitalization (21 days vs. 13 days, *P* < 0.001) and higher in hospital mortality (8.5% vs. 0.2%, *P* < 0.001).

Overall, 86 patients (7.6%; 95% CI, 6.1-9.2%) developed SAP and 47 patients (4.3%; 95% CI, 3.0-5.3%) developed GIB during hospitalization.

**Table 1:** Baseline characteristics in relation to the status of gastrointestinal bleeding

	Overall (*N* = 1129)	Without GIB (*N* = 1082)	With GIB (*N* = 47)	*P* value
Demographics				
Age, mean (SD)	58.6 ± 12.5	58.6 ± 12.5	60.6 ± 11.4	0.276
Males, n (%)	899 (79.6%)	862 (79.7%)	37 (78.7%)	0.875
Stroke risk factors, n (%)				
Hypertension	755 (66.9%)	720 (66.5%)	35 (74.5%)	0.259
Diabetes mellitus	341 (30.2%)	325 (30.0%)	16 (34.0%)	0.558
Dyslipidemia	206 (18.2%)	202 (18.7%)	4 (8.5%)	0.078
Atrial fibrillation	69 (6.1%)	61 (5.6%)	8 (17.0%)	0.001
Coronary artery disease	151 (13.4%)	145 (13.4%)	6 (12.8%)	0.900
History of stroke/TIA	265 (23.5%)	251(23.2%)	14 (29.8%)	0.297
Current smoking	628 (55.6%)	601 (55.5%)	27 (57.4%)	0.797
Comorbidities, n (%)				
Valvular heart disease	10(0.9%)	9 (0.8%)	1(2.1%)	0.353
COPD	26 (2.3%)	24 (2.2%)	2 (4.3%)	0.362
Peripheral artery disease	8 (0.7%)	7 (0.6%)	1 (2.1%)	0.236
Hepatic cirrhosis	17 (1.5%)	14 (1.3%)	3 (6.4%)	0.005
Peptic ulcer	21 (1.9%)	20 (1.8%)	1 (2.1%)	0.890
Renal failure	7 (0.6%)	6 (0.6%)	1 (2.1%)	0.179
Arthritis	16 (1.4%)	15 (1.4%)	1 (2.1%)	0.674
Cancer	14 (1.2%)	13 (1.2%)	1 (2.1%)	0.574
Medication				
Antiplatelet drug	144 (12.8%)	137 (12.7%)	7 (14.9%)	0.653
Anticoagulants	20 (1.8%)	19 (1.8%)	1 (2.1%)	0.850
In-hospital interventions				
Antiplatelet therapy within 48 h	1098 (97.3%)	1057 (97.7%)	41 (87.2%)	< 0.001
rTPA	121 (10.8%)	116 (10.8%)	5 (10.6%)	0.977
Admission NIHSS score, median (IQR)	4 (2–8)	4 (2–8)	13 (7–20)	< 0.001
Infarct location				
Posterior circulation infarct, n (%)	481 (42.6%)	454 (40.2%)	27 (57.45%)	0.049
Pneumonia, n (%)	86 (7.6%)	62 (5.7%)	24 (51.1%)	< 0.001
Length of hospitalization (days), median (IQR)	14 (11–16)	13 (11–16)	21 (13–26)	< 0.001
Mortality	6 (0.5%)	2 (0.2%)	4 (8.5%)	< 0.001

TIA indicated transient ischemic attack; COPD, chronic obstructive pulmonary disease; GIB, gastrointestinal bleeding; mRS, modified Rankin Scale; rTPA, recombinant tissue plasminogen activator; NIHSS, National Institutes of Health Stroke Scale; IQR, interquartile range

### Association between SAP and GIB

The unadjusted regression model indicated that SAP was significantly associated with the development of GIB after AIS (OR = 17.17; 95% CI, 9.17–32.13; *P* < 0.001). After adjusted for age, gender, and other potential confounders, SAP was still significantly associated with the development of GIB from model 2 to model 4 (Table 2). In the model 4, the odds ratio of development of GIB in the patients with SAP was 5.10 (95% CI, 2.01–12.95) compared with patients without SAP.

**Table 2:** Odds ratio (OR) for gastrointestinal bleeding after ischemic stroke

	OR (95% CI)	*P* Value
Model 1	17.17 (9.17–32.13)	< 0.001
Model 2	20.37 (10.22–40.62)	< 0.001
Model 3	7.49 (3.23–17.34)	< 0.001
Model 4	5.10 (2.01–12.95)	< 0.001

Model 1 is crude model; Mode 2 adjusted for age, gender, hypertension, diabetes, dyslipidemia, atrial fibrillation, smoking, prestroke dependence (modified Rankin Scale score ≥ 3); Model 3 adjusted for model 2 plus National Institutes of Health Stroke Scale, and comorbidities (hepatic cirrhosis, peptic ulcer, cancer); Model 4 adjusted for model 3 plus pre-hospital antiplatelet drug, pre-hospital anticoagulant, antiplatelet therapy within 48 h, intravenous thrombolysis, and length of hospitalization. OR indicates odds ratio; CI, confidence interval

### Temporal sequence of SAP and GIB after acute ischemic stroke

**Fig. 1:**
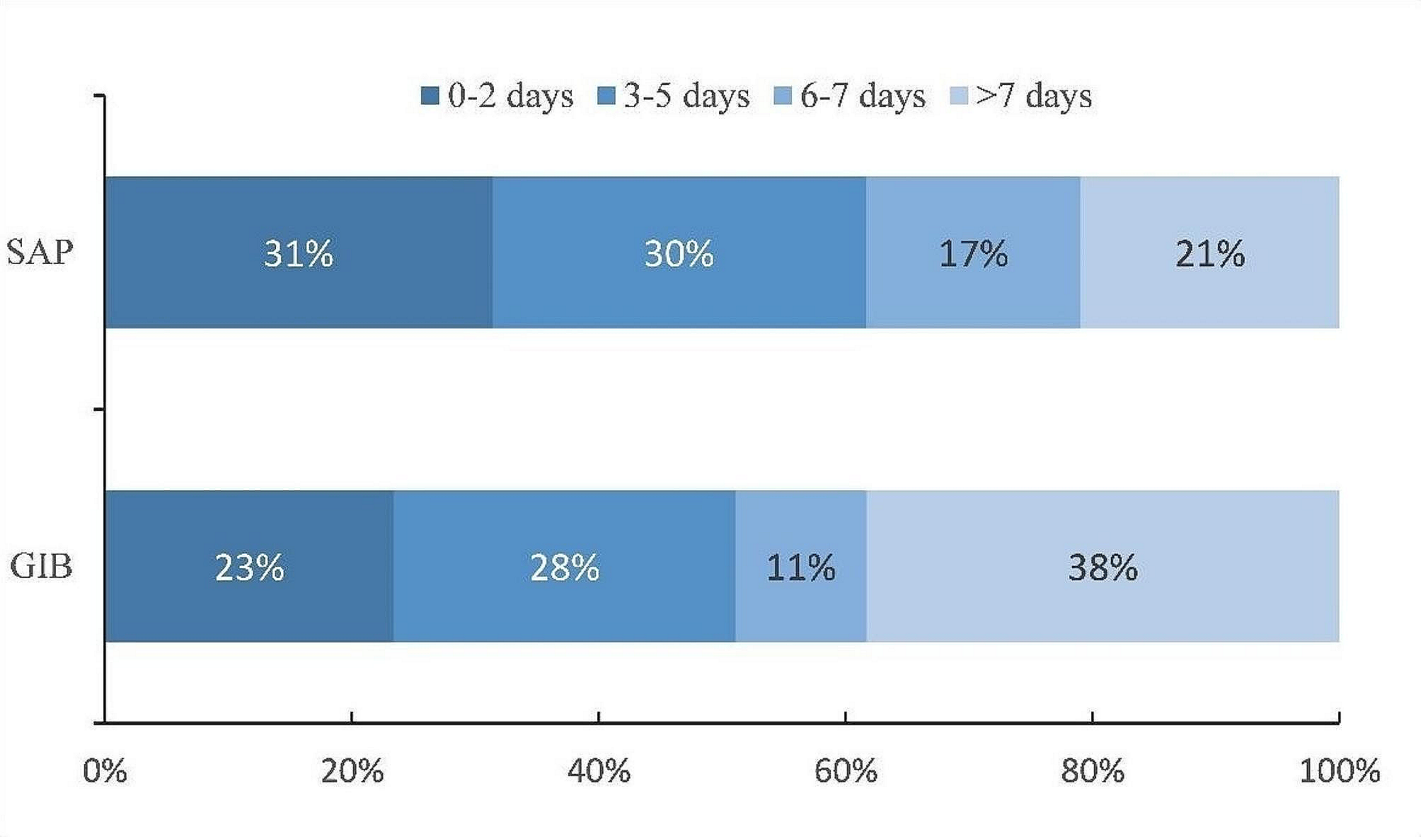
The proportion distribution of days from ischemic stroke onset to complications diagnosis in patients with pneumonia and gastrointestinal bleeding. SAP, stroke-associated pneumonia; GIB, gastrointestinal bleeding

Within the first 48 h from stroke onset, 27 (31.4%) of the patients with SAP and 11 (23.4%) of the patients with GIB were diagnosed. Up to 7 days from stroke onset, 79.1% and 61.7% of patients with PNE and GIB, respectively, was diagnosed with corresponding complications (Fig. 1).

**Fig. 2:**
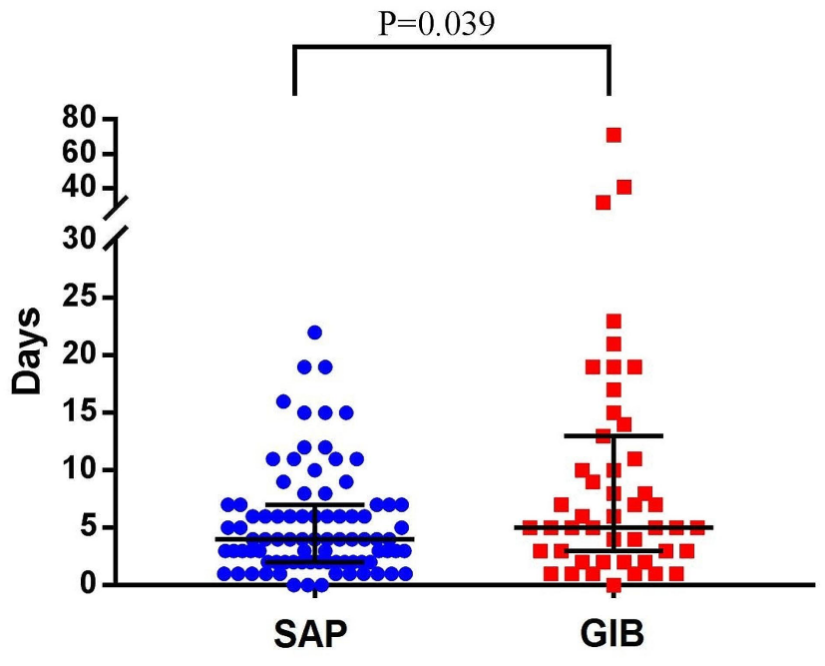
The distribution of days from ischemic stroke onset to pneumonia and gastrointestinal bleeding diagnosis. SAP, stroke-associated pneumonia; GIB, gastrointestinal bleeding

The distribution of timing (days) from stroke onset to diagnosis of SAP and GIB are shown in Fig. 2. Overall, the median time from stroke onset to diagnosis of SAP was significantly shorter than that of GIB after acute stroke (4 days vs.5 days; *P* = 0.039). When the patients were stratified by severity of stroke and infarct location, only in patients with NIHSS ≥ 16 the significant difference was observed (Table 3).

**Table 3:** Days from stroke onset to diagnosis of pneumonia and gastrointestinal bleeding stratified by NIHSS and infarct location

	Days from stroke onset to diagnosis of SAP, median (IQR)	Days from stroke onset to diagnosis of GIB, median (IQR)	*p*
NIHSS < 16	6 (3–8)	5 (3–10)	0.784
NIHSS ≥ 16	3 (1–4)	6 (3–15)	0.004
Posterior circulation infarct	5 (2–15)	4 (2–7)	0.110
Non-posterior circulation infarct	4 (2–7)	5.5 (3-10.5)	0.154

NIHSS indicated National Institutes of Health Stroke Scale; SAP, stroke-associated pneumonia, GIB, gastrointestinal bleeding

## Discussion

In the present study, the incidence of SAP and GIB after stroke was 7.6% and 4.3% during hospitalization. We found that SAP was associated with GIB after ischemic stroke and the time interval from stroke onset to SAP diagnosis was earlier than that of GIB. Our findings suggested that SAP may be a risk factor or risk marker for GIB in patients with ischemic stroke.

Previous studies have identified several risk factors of GIB after ischemic stroke, such as age, previous history of peptic ulcer, severe stroke, hypertension, hepatic cirrhosis, renal failure, and cancer [11, 18, 19]. In the present study, the mean age was older and the proportion of previous peptic ulcer, hypertension, renal failure and cancer was higher in GIB patients compared with non-GIB patients. However, the univariate analysis did not reveal any statistically significant difference in these factors between GIB group and non-GIB group. This may due to small sample size and population characteristics. However, there were few studies investigated the association between SAP and GIB in patients with stroke. Cook, D. J. et al. [20] analyzed 2252 patients admitted to intensive care units and found respiratory failure was one of the strong independent risk factors for GIB. Rumalla, K. et al. [12] examined the association of GIB with other in-hospital complications in patients with AIS and indicated that GIB was significantly associated with SAP. This was consistent with our present study. Additionally, in our previous study, we analyzed 11 common stroke-associated medical complications and observed SAP was strongly associated with the development of GIB after AIS and hemorrhagic stroke [15]. However, all of these studies failed to explore the temporal sequence of SAP and GIB. Stroke can suppress systemic immune response namely immunosuppression, leading to more vulnerable to infection after stroke, such as pneumonia [21, 22]. However, prophylactic antibiotic treatment cannot significantly reduce the risk of SAP, although it may lower the risk of total and urinary tract infections [21]. Recent research suggested that stroke and myocardial infarction can cause a significant loss of intestinal B and T cells through the release of neutrophil extracellular traps (NETs), and DNase-I therapy may be a potential treatment for patients with stroke [22]. The pathophysiological mechanism through which patients after stroke developed GIB was not entirely understood. Stress, antiplatelet use, systemic inflammation, and oxidative stress have been proposed to illustrate the mechanism of post-stroke GIB [23]. The axis between the central nervous system and gastrointestinal system may be interrupted because of cerebral ischemia, which in turn lead to the gastrointestinal symptoms such as GIB [24]. In a porcine model of peritonitis and hemorrhage, gut blood flow and intramucosal pH were all decreased and the intramucosal pH decrease preceded that of blood flow [25]. A decreasing intramucosal pH was associated with an increased oxygen extraction ratio, which was not sufficient to maintain aerobic metabolism [25]. In critical illness patients, Mutlu, Go˙khan M. et al. [26]. suggested mechanical ventilation (MV) may potentiate the adverse effects of an underlying critical illness and worsen gastrointestinal pathophysiology. They proposed a mechanism for the development of gastrointestinal complications during MV. On the one hand, MV can affect splanchnic blood flow. On the other hand, MV can increase the release of proinflammatory mediators [27]. The proinflammatory mediators have an effect on splanchnic hypoperfusion and may impair intestinal smooth muscle function [28, 29]. Lung injuries or acute respiratory distress syndrome could induce multiple organ dysfunction syndromes by releasing inflammatory mediators into blood [30, 31]. However, the relationship between inflammatory response and SAP and their effect on GIB are complex. The coexistence of inflammatory response after stroke makes it impossible to illustrate whether the SAP directly contributes to the GIB. In our previous study, we hypothesized four stages to explain pathophysiological mechanisms for the interrelationship between SAP and non-SAP complications [15]. Based on this evidence, we suggested a potentially critical role for SAP in the initiation and propagation of systemic inflammatory response which may lead to GIB.

While we are unable to conclude the direct causal relationship between SAP and GIB, the association and temporal sequence for SAP and GIB can guide further research. The pathophysiological mechanism for SAP contributing to GIB after stroke should be studied further. Additionally, it seems reasonable to pay additional attention to patients with SAP after stroke and the prophylactic interventions can be taken to prevent GIB. It was supposed that prophylactic acid suppression therapies, such as H_2_ antagonists and proton pump inhibitor therapies, may reduce the risk of GIB [11]. However, the evidence on prophylactic medication to prevent GIB in stroke patients was limited. Further clinical studies were needed to evaluate the clinical efficacy of prophylactic acid suppression therapies on GIB after stroke.

To our knowledge, this is the first time to examine the temporal sequence of SAP and GIB in patients with AIS. Our study has several limitations. Firstly, the etiology of GIB was not recorded. Although, previous studies revealed that the bleeding mainly originated in upper gastrointestinal tracts and more than one fifth could not identify despite endoscopic examination [14, 32]. Secondly, the details about severity for SAP and GIB were not reported in consequence of unable to explore whether the dose-response relationship existed. Thirdly, we failed to obtain the information on medication to prevent stress ulcers. Fourthly, since the clinical symptoms of GIB was insidious, it may be not accuracy to define the timing of GIB in terms of diagnosis establishment. The occurrence of disease is a continuous process and it is practicable to the diagnosis timing to define the occurrence of disease. In order to reduce this bias, we used multiple evidence, including clinical, laboratory or radiographic evidence to define the timing of GIB occurred.

## Conclusions

In conclusion, GIB after stroke was associated with SAP and the median timing from stroke onset to SAP diagnosis was earlier than that of GIB diagnosis. When the patients after stroke were diagnosed with SAP, it should be paid more attention and take measures to prevent GIB.

## Data Availability

The data that support the findings of this study are available from the corresponding author upon reasonable request.

## Abbreviations

AIS: Acute ischemic stroke
SAP: Stroke-associated pneumonia
GIB: Gastrointestinal bleeding
NIHSS: National Institutes of Health Stroke Scale
mRS: Modified Rankin Scale score
TIA: Transient ischemic attack
COPD: Chronic obstructive pulmonary disease
rTPA: Recombinant tissue plasminogen activator
IQR: Interquartile range
OR: Odds ratio
CI: Confidence interval
MV: Mechanical ventilation
